# Alterations in regulators of the renal-bone axis, inflammation and iron status in older people with early renal impairment and the effect of vitamin D supplementation

**DOI:** 10.1093/ageing/afae096

**Published:** 2024-05-20

**Authors:** Marilena Christodoulou, Terence J Aspray, Isabelle Piec, William D Fraser, Inez Schoenmakers, VDOP Trial group, Terry J Aspray, Roger M Francis, Elaine McColl, Thomas Chadwick, Ann Prentice, Inez Schoenmakers

**Affiliations:** University of East Anglia, Norwich Medical School, Norwich, UK; Freeman Hospital, Bone Clinic, University of Newcastle upon Tyne, Newcastle upon Tyne, UK; University of East Anglia, Norwich Medical School, Norwich, UK; University of East Anglia, Norwich Medical School, Norwich, UK; Clinical Biochemistry, Department of Laboratory Medicine and Department of Diabetes and Endocrinology, Norfolk and Norwich University Hospital NHS Foundation Trust, Norwich, UK; University of East Anglia, Norwich Medical School, Norwich, UK; MRC Human Nutrition Research, Cambridge, UK

**Keywords:** renal impairment, fibroblast growth factor 23, vitamin D, inflammation markers, iron status, older people

## Abstract

**Context:**

Chronic kidney disease (CKD) leads to alterations in fibroblast growth factor 23 (FGF23) and the renal-bone axis. This may be partly driven by altered inflammation and iron status. Vitamin D supplementation may reduce inflammation.

**Objective and methods:**

Older adults with early CKD (estimated glomerular filtration rate (eGFR) 30–60 ml/min/1.73 m^2^; CKDG3a/b; *n* = 35) or normal renal function (eGFR >90 ml/min/1.73 m^2^; CKDG1; *n* = 35) received 12,000, 24,000 or 48,000 IU D_3_/month for 1 year. Markers of the renal-bone axis, inflammation and iron status were investigated pre- and post-supplementation. Predictors of c-terminal and intact FGF23 (cFGF23; iFGF23) were identified by univariate and multivariate regression.

**Results:**

Pre-supplementation, comparing CKDG3a/b to CKDG1, plasma cFGF23, iFGF23, PTH, sclerostin and TNFα were significantly higher and Klotho, 1,25-dihydroxyvitamin D and iron were lower. Post-supplementation, only cFGF23, 25(OH)D and IL6 differed between groups. The response to supplementation differed between eGFR groups. Only in the CKDG1 group, phosphate decreased, cFGF23, iFGF23 and procollagen type I N-propeptide increased. In the CKDG3a/b group, TNFα significantly decreased, and iron increased. Plasma 25(OH)D and IL10 increased, and carboxy-terminal collagen crosslinks decreased in both groups. In univariate models cFGF23 and iFGF23 were predicted by eGFR and regulators of calcium and phosphate metabolism at both time points; IL6 predicted cFGF23 (post-supplementation) and iFGF23 (pre-supplementation) in univariate models. Hepcidin predicted post-supplementation cFGF23 in multivariate models with eGFR.

**Conclusion:**

Alterations in regulators of the renal-bone axis, inflammation and iron status were found in early CKD. The response to vitamin D_3_ supplementation differed between eGFR groups. Plasma IL6 predicted both cFGF23 and iFGF23 and hepcidin predicted cFGF23.

## Key points

- Differences in regulators of the renal-bone axis, inflammation and iron status were detected between older people with early renal impairment (CKDG3a/b) and counterparts with normal renal function.
- The response to vitamin D supplementation depended on renal function, reducing tumour necrosis factor alpha (TNFα) and increasing iron status with early renal impairment (CKDG3a/b) and increasing fibroblast growth factor 23 (FGF23) in those with normal renal function.
- Predictors of intact and c-terminal fibroblast growth factor 23 (FGF23) were eGFR, regulators of calcium and phosphate metabolism and interleukin 6 (IL6).

## Introduction

Renal impairment results in alterations in regulators of the renal-bone axis through multiple pathways. Fibroblast growth factor 23 (FGF23) and inflammation increase and iron status may decrease [1, 2]. In healthy people, plasma phosphate (P), 1,25-dihydroxyvitamin D (1,25(OH)_2_D) and PTH are the main regulators of plasma FGF23, but from early stages of chronic kidney disease (CKD), FGF23 increases before an increase in plasma P is observed [3]. It has been suggested that increased inflammation and iron deficiency play a role through alteration of FGF23 transcription and post-translational modification [4]. The pro-inflammatory cytokines tumour necrosis factor alpha (TNFα) and interleukin 6 (IL6) and iron deficiency have been shown to increase osteocytic FGF23 transcription and cleavage, resulting in increased plasma concentrations of particularly c-terminal FGF23 (cFGF23) and to a lesser extent intact FGF23 (iFGF23) [4, 5]. Also hepcidin, the main hormonal regulator of systemic iron homeostasis may indirectly influence FGF23 concentrations since hepcidin increases in response to inflammation and inhibits the incorporation of iron into erythrocytes. In reverse, cFGF23 has recently been shown to regulate iron metabolism [4, 6–8].

High concentrations of FGF23, increased inflammation and iron deficiency have been reported to be associated with increased risk of CVD and progression of CKD, poor skeletal integrity, low bone mineral density (BMD) and bone loss [9–11] and alterations of sclerostin (SOST) and RANKL, regulators of Wnt signalling [12, 13]. This suggests that these factors may mediate CKD associated mineral and bone disorder (CKD-MBD).

Also vitamin D status may play a role. Vitamin D deficiency, measured as the plasma concentration of 25-hydroxy vitamin D (25(OH)D), is common in CKD [14]. Negative associations between 25(OH)D concentrations and c-reactive protein (CRP) [15], pro-inflammatory cytokines, particularly IL6 [16–20] and iron status [21–23] have been reported in diverse populations and patient groups but evidence is conflicting [24–28]. Vitamin D supplementation may decrease inflammatory cytokines and hepcidin and through this, increase erythropoiesis, but human studies are limited and have provided mixed results [8, 21, 29–31]. On the basis of these findings, it may be speculated that vitamin D supplementation may downregulate FGF23. However, we and others earlier reported that vitamin D supplementation was associated with an increase, rather than a decrease in iFGF23 and cFGF23 [14, 32, 33].

Although changes in regulators of the renal-bone axis may occur from early renal impairment, most people are asymptomatic and not diagnosed until their estimated glomerular filtration rate (eGFR) is less than 30 ml/min/1.73 m^2^ [34–37]. For the development of strategies to promote healthy ageing and for prevention of the progression of CKD and development of CKD-MBD, understanding changes in regulators of bone and mineral metabolism from early renal impairment is crucial. We investigated the influence of early renal impairment on regulators and markers of the renal-bone axis, inflammation and iron status and their response to vitamin D_3_ supplementation. Predictors of cFGF23 and iFGF23 were investigated before and after supplementation. For this study, participants of a double-blind randomised dose–range intervention trial with vitamin D_3_ were selected on basis of their eGFR according to the Modification of Diet in Renal Disease-4 variable equation (MDRD-4), generating two subgroups with an eGFR 30–60 ml/min/1.73m^2^ (CKDG3a and G3b) and eGFR >90 ml/min/1.73m^2^ (CKDG1; normal renal function).

## Materials and methods

### Study design

This study was a *post-hoc secondary analyses* of plasma samples and data collected as part of the vitamin D_3_ supplementation in older people (VDOP) randomised controlled trial [38] (ISRCTN35648481). In brief, this RCT included 379 ambulatory, community dwelling adults aged ≥70 years (48% women) from the northeast of England. Those with known CKD or MDRD-4 based eGFR <30 ml/min/1.73m^2^ at pre-screening were excluded. Participants were randomly allocated to three groups, supplemented with three different oral dosages of vitamin D_3_ [12,000 international units (IU), 24,000 IU or 48,000 IU] (Merck Sereno GmbH) given once a month for 1 year. The dose of 12,000 IU/m corresponds to the UK reference nutrient intake (RNI) and represents standard care. More details of the study design, methods and primary outcomes were previously described [38, 39]. From this cohort, participants were selected if their eGFR at both time points (baseline and 12 months) was <60 ml/min/1.73 m^2^ based on MDRD-4 and were compared with to a group of participants with an eGFR >90 ml/min/1.73 m^2^. Additionally, only those with samples available at both time points were selected and haemolytic samples were not used. This resulted in *n* = 70 sets of data, with *n* = 35 participants in each eGFR group.

The analyses were explorative and secondary and were not pre-specified in the original trial design and analyses plan. Results for plasma concentrations of 25(OH)D, intact-parathyroid hormone (iPTH), BMD and Wnt signalling markers in the full cohort, without considering eGFR were earlier reported [33, 38].

The study was conducted in accordance with guidelines laid down in the Declaration of Helsinki. All participants provided written informed consent. The Tyne & Wear South Research Ethics Committee (REC 12/NE/0050) approved the study.

### Measurements

Methods for measurement of BMD, height and weight, collection and processing of early morning fasting blood samples at baseline and after 12 months of supplementation, and biochemical analyses were provided elsewhere [33, 38]. Sample collection and processing methods were checked for suitability for measurement of iron status and other markers included in this study.

In brief, measurements and analytical methods were as follows (with methods in brackets):

Regulators of the renal-bone axis: plasma creatinine, calcium, albumin, phosphate (Cobas, Roche Diagnostics); iPTH (Immulite 2000, SIEMENS); iFGF23 and cFGF23 (Immutopics); αKlotho (IBL international); 25(OH)D (LC–MS/MS); 1,25(OH)_2_D (DiaSorin, Liaison XL). Inflammation and iron status and markers: C-reactive protein (CRP); high sensitivity) IL-6 and iron (Cobas, Roche Diagnostics); TNFα, IL-10 (high sensitivity) and hepcidin (R&D systems Bio-Techne). Bone turnover and Wnt signalling markers: bone specific alkaline phosphatase (BAP) (DiaSorin, Liaison); carboxy-terminal collagen crosslinks (CTX) (Immunodiagnostic Systems); procollagen 1 intact N-terminal propeptide (P1NP) (UniQ, RIA); SOST, Dickkopf-related Protein 1 (DKK1); Osteoprotegerin (OPG) and soluble RANKL (sRANKL) (Biomedica).

All assays were performed in duplicate except for phosphate, iPTH, 1,25(OH)_2_D, CRP, IL-6 and iron on basis of consistent performance with intra and inter-assay coefficients of variation (CV) <4%. Assay performance was monitored using kit and in-house controls and under strict standardisation according to ISO 9001:2000 or following Good Laboratory Practice. External Quality assurance of 25(OH)D and iPTH assays are performed as part of the Vitamin D External Quality Assessment Scheme (www.deqas.org) and the National External Quality Assessment Scheme (www.ukneqas.org.uk). Measurements of 25(OH)D were harmonised against NIST standards as part of the Vitamin D harmonisation program [38].

Calculation of eGFR was according to the MDRD-4 algorithm without race, since all participants were White. Analyses were also conducted with the CKD Epidemiology Collaboration (CKD-EPI) algorithm with creatinine and without race [40] and provided very similar results (not presented).

### Statistical analysis

This research aimed to test:

(1) Differences in FGF23 and markers of inflammation and iron status and regulators of bone metabolism between eGFR categories before and after 12 months of vitamin D_3_ supplementation.
(2) The effect of vitamin D_3_ supplementation by eGFR group.
(3) Predictors of cFGF23 and iFGF23.

A sample size calculation for this secondary analysis was based on a detectable difference in IL6 and TNFα between eGFR categories (aim 1) and between time points (aim 2) with a significance level of 5% and 90% power. Data from the MrOs study were utilised, which included older male participants and similar laboratory methods as used in this study [24]. With a sample size of *n* = 35 for each eGFR category, the detectable difference between eGFR categories is 50–60%, and the detectable effect size between time points is 25–30%. For the latter power calculation, it was assumed that the within-subject variation is approximately half the size of the between subject estimate [33].

Between eGFR group differences pre- and post-supplementation were tested by ANCOVA, with supplementation group as a covariate for 12 months data (aim 1). The response to supplementation within each eGFR category was tested by ANCOVA with supplementation group as a covariate, which was removed if non-significant (aim 2). Correction for repeated testing was not deemed appropriate for this explorative analysis, as any finding will require confirmation in RCTs specifically designed and powered for respective outcomes.

Predictors of cFGF23 and iFGF23 at each time-point (aim 3) were identified by univariate linear regression analysis with eGFR as a continuous variable (i.e. including data for both eGFR categories). Linearity of associations were checked visually. Subsequent multivariate linear regression included variables with a p-value <0.2 in univariate analyses (Tables 2 and 3), followed by hierarchical elimination of non-significant variables. Co-linearity of independent variables (*r* > 0.6) was checked prior to inclusion in multivariate models. Since the assay for cFGF23 recognises both c-terminal fragments and intact FGF23, plasma concentrations of cFGF23 and iFGF23 were expected to be highly correlated. Therefore, multivariate analyses were conducted with and without inclusion of cFGF23 or iFGF23 as independent variables. Potential predictors cFGF23 and iFGF23 were selected *a priori* on the basis of theoretical biological mechanisms. Regression analysis with markers of bone density and metabolism were not conducted as these were not considered as primary regulators of FGF23.

All outcomes were assessed for normality (defined as a posterior distribution skewness <2 or > −2) and visual inspection of histograms. Non-normally distributed variables were converted to natural logarithm values (LN) and checked again for normality. One extreme outlier was identified for TNFα and was excluded. For normally distributed data, results are expressed as mean (SD) and for skewed data, as median [interquartile range (IQR)].

IBM® SPSS® Statistics Version 28 software was used.

## Results

### Differences in biomarkers between eGFR categories

Median eGFR was 51.0 [IQR: 45.8–53.8] ml/min/1.73 m^2^ (CKDG3a: *n* = 25; CKDG3b: *n* = 10) and 95.7 [92.1–102.8] for the CKDG3a/b and G1 group, respectively. Age was significantly higher in the group with CKD3a/b; gender and body mass index did not differ (Table 1).

**Table 1:** Comparisons between groups categorised on the basis of eGFR (CKD3a/b and CKDG1) at baseline and 12 months^a^

Characteristics^a^	eGFR < 60 ml/min/1.73 m^2^ (*n* = 35)^b^				eGFR > 90 mL/min/1.73 m^2^ (*n* = 35)^c^			
	Baseline		12 months		Baseline		12 months	
Men/women	18/17		–		20/15		–	
Age (years)	76.9	(4.1)	–		–	(4.1)^*^^*^^*^	-	
BMI (kg/m^2^)	27.9	(4.5)	–		27.6	(4.5)	–	
MDRD-4 eGFR (mL/min/1.73 m^2^)^d^	51.0 [45.8–53.8]				95.7 [92.1–102.8]^*^^*^^*^			
MDRD-4 eGFR (mL/min/1.73 m^2^)	50.0 [44.8–53.1]		51.0 [44.6–54.4]		97.5 [93.3–103.0]		94.5 [89.6–105.0]	
**Markers of calcium, phosphate and vitamin D metabolism**								
Calcium_ALB_ (mmol/l)	2.2	(0.1)	2.2	(0.1)	2.2	[2.2–2.3]	2.2	(0.1)
Phosphate (mmol/l)	0.81	(0.2)	0.83	(0.2)	0.88	(0.17)^*^^*^^*^^*^	0.78	(0.16)
iPTH (pg/ml)	59.9	(27.1)	52.7	(23.9)	42.9	[27.9–67.8]^*^	42.3	(22.6)
iFGF23 (pg/ml)	69.4	[49.7–78.4]	78.4	[54.9–91.5]	53.4	(19.0)^*^^,^^*^^*^^*^^*^	57.1	[48.1–68.2]
cFGF23 (RU/ml)	81.9	[71.0–154.6]	112.0	[84.8–139.8]	58.2	(18.5)^*^^*^^,^^*^^*^^*^^*^	61.1	[53.4–74.6]^*^
Klotho (pg/ml)	436	(102)	471.1	(109)	509	[392–643]^*^	507.4	[403–730]
25(OH)D (nmol/l)	30.0	[21.8–39.0]^*^^*^^*^^*^	73.5	(20.3)	39.9	(21.2)^*^^*^^*^^*^	61.5	(18.7)^*^
1,25(OH)_2_D (pmol/l)	71.2	[56.1–102.0]	82.4	(29.6)	111.5	(30.9)^*^^*^	112.0	(31.6)
**Iron status and inflammation markers**								
CRP (nmol/l)	18.8	[8.1–35.5]	18.3	[8.2–39.2]	15.5	[7.5–27.7]	12.8	[4.9–27.9]
TNFα (pg/ml)	9.4	[7.8–11.1]^*^^*^^*^^*^	6.5	[5.1–12.0]	6.8	[5.8–9.8]^*^	7.0	[4.2–8.8]
IL6 (pg/ml)	2.54	[0.75–4.61]	2.93	[1.78–5.85]	0.75	[0.75–2.90]	0.75	[0.75–2.80]^*^
IL10 (pg/ml)	0.33	[0.02–0.61]^*^^*^^*^^*^	0.52	[0.38–1.00]	0.18	[0.02–0.60]^*^^*^^*^^*^	0.69	[0.52–0.81]
Iron (μmol/l)	12.6	(6.4)^*^^*^^*^^*^	16.2	(6.4)	16.1	(6.0)^*^	18.0	(5.8)
Hepcidin (ng/ml)	24.7	(16.0)	20.2	(14.1)	19.7	[11.9–31.6]	19.6	11.2
**Bone turnover and Wnt signalling markers**								
Hip BMD (g/m^2^)	1.01	(0.19)	1.00	(0.19)	0.95	(0.14)^*^^*^^*^^*^	0.94	(0.15)
BAP (μg/l)	9.5	[7.4–11.3]	9.8	(3.1)	11.4	(3.6)	11.6	[8.3–14.2]
CTX (ng/ml)	0.41	[0.29–0.58]^*^^*^^*^^*^	0.36	(0.15)	0.37	[0.28–0.53]^*^^*^^*^^*^	0.34	[0.26–0.42]
PINP (μg/l)	41.4	[29.1–53.5]	40.3	[31.8–50.7]	38.3	[26.8–46.0]^*^^*^^*^^*^	40.0	[32.7–47.5]
SOST (pmol/l)	61.4	(22.6)	62.3	(24.0)	41.5	(16.7)^*^^*^	41.9	(18.6)
DKK1 (pmol/l)	25.0	(12.1)	32.9	(15.7)	30.6	(13.3)	40.9	(19.2)
OPG (pmol/l)	5.40	[4.39–6.29]	5.45	[4.45–6.61]	5.25	(1.86)	5.26	(1.61)
sRANKL (pmol/l)	0.12	[0.09–0.14]	0.11	[0.07–0.21]	0.14	(0.09)	0.13	[0.07–0.22]

BMD, bone mineral density; BAP, bone specific alkaline phosphatase; CTX, carboxy-terminal collagen crosslinks; calcium_ALB_, albumin adjusted calcium; cFGF23 and iFGF23, c-terminal and intact fibroblast growth factor-23; CRP, C-reactive protein; DKK1, Dickkopf-related protein 1; eGFR, estimated glomerular filtration rate; 25(OH)D, 25-hydroxy vitamin D; 1,25(OH)_2_D, 1,25-dihydroxy vitamin D; iPTH, intact-parathyroid hormone; IL6, interleukin 6; IL10, interleukin 10; OPG, osteoprotegerin; PINP, procollagen 1 intact N-terminal; SOST, sclerostin; sRANKL, soluble receptor activator of nuclear factor-κB ligand; TNFα, tumour necrosis factor alpha.

^a^For normally distributed data, results are expressed as mean (SD); for skewed data, results are expressed as median [interquartile range (IQR)].

^b^Arm: 12000 IU *n* = 4; 24,000 IU *n* = 13; 48,000 IU *n* = 18.

^c^Arm: 12000 IU *n* = 8; 24,000 IU *n* = 16; 48,000 IU *n* = 11.

^d^Mean of pre- and post-supplementation values.

^*^ANCOVA significant difference *P* < 0.05 between eGFR groups

^**^ANCOVA significant difference *P* < 0.001 between eGFR groups

^***^Independent *t*-test significant difference *P* < 0.05 between eGFR groups

^****^ANCOVA significant difference *P* < 0.05 between pre- and post-supplementation values within eGFR groups

At baseline, compared to the group with normal renal function, plasma PTH, cFGF23, iFGF23 were higher, klotho and 1,25(OH)_2_D were lower in the group CKDG3a/b. Of the inflammation markers, the pro-inflammatory cytokine TNFα was significantly higher and IL6 near significantly (*P* = 0.075) higher in the group with CKDG3a/b, but plasma CRP and IL10 did not differ. Plasma iron was lower, but there was no difference in the hepcidin concentration between groups. SOST was higher in the CKDG3a/b group, but no differences were found in other markers of the Wnt signalling pathway and bone density and remodelling (Table 1).

Post vitamin D_3_ supplementation, cFGF23 concentrations remained significantly different between eGFR groups. Post-supplementation 25(OH)D was higher in the group with CKDG3a/b. The markers of inflammation, CRP, TNFα and IL10 were not different between groups, but the pro-inflammatory cytokine IL6 was significantly higher in the group with CKDG3a/b. There were no group differences in plasma iron and hepcidin. There were also no between eGFR group differences in markers of Wnt signalling and bone density and remodelling (Table 1).

### Supplementation effect within each eGFR group

Estimated GFR did not change. There were differences in the response to supplementation between eGFR groups. In the group with CKDG3a/b, plasma P, iFGF23 and cFGF23 were unchanged, whereas in the group with normal renal function plasma P decreased and both cFGF23 and iFGF23 increased (both *P* < 0.01). Plasma 25(OH)D increased in both groups and the remainder markers of calcium and phosphate metabolism did not significantly change in either group.

In the group with CKDG3a/b, TNFα decreased and IL10 increased. Plasma CRP and IL6 did not change. Plasma iron increased, while hepcidin was unchanged. The bone resorption marker CTX decreased, but BMD, BAP, P1NP and markers of Wnt signalling were unchanged. In the group with normal renal function, no changes were observed in markers of inflammation and iron status, except for an increase in IL10. CTX and BMD decreased and P1NP increased. BAP and markers of Wnt signalling were unchanged (Table 1).

Supplementation group was significant for cFGF23, 25(OH)D and IL6.

### Predictors of cFGF23 and iFGF23

At baseline, predictors of cFGF23 were eGFR, iFGF23, PTH and 1,25(OH)_2_D, but no significant associations were found with markers of inflammation and iron status in univariate analyses, although a tendency of significance was detected for plasma iron (*P* = 0.076) (Table 2). In a multivariate model, only eGFR remained significant (total *R*^2^ = 19%) (Table 3).

**Table 2:** Predictors of c-terminal and intact FGF23 at baseline and 12 months^a^ in univariate regression models

	Baseline		12 months	
	β-coefficient (SE)	*P* value	β-coefficient (SE)	*P* value
cFGF23 (RU/ml)				
eGFR (ml/min/1.73 m^2^)	**−1.70 (0.266)**	**<0.001**	**−1.13 (0.269)**	**<0.001**
Calcium_ALB_ (mmol/l)	24.0 (131.775)	0.856	**293.6 (113.670)**	**0.012**
Phosphate (mmol/l)	−74.6 (41.539)	0.077	53.1 (46.710)	0.260
iFGF23 (pg/ml)	**0.72 (0.261)**	**0.007**	**0.92 (0.226)**	**<0.001**
Klotho (pg/ml)	−0.037 (0.035)	0.294	−0.051 (0.037)	0.167
iPTH (pg/ml)	**0.66 (0.304)**	**0.033**	**0.67 (0.327)**	**0.045**
25(OH)D (nmol/l)	−0.56 (0.413)	0.178	0.15 (0.392)	0.711
1,25(OH)_2_D (pmol/l)	**−0.56 (0.220)**	**0.013**	**−0.50 (0.228)**	**0.030**
CRP (nmol/l)	0.143 (0.128)	0.269	0.309 (0.168)	0.070
TNFa (pg/ml)	1.23 (2.102)	0.561	1.26 (1.520)	0.410
IL6 (pg/ml)	1.32 (1.690)	0.437	**3.54 (1.764)**	**0.049**
IL10 (pg/ml)	−9.33 (18.806)	0.622	2.99 (4.434)	0.502
Iron (μmol/l)	−2.26 (1.253)	0.076	−0.67 (1.295)	0.599
Hepcidin (ng/ml)	<−0.001 (0.001)	0.513	−0.001 (0.001)	0.077
**iFGF23 (pg/ml)**				
eGFR (ml/min/1.73 m^2^)	**−0.38 (0.122)**	**0.003**	**−0.45 (0.135)**	**0.002**
Calcium_ALB_ (mmol/l)	**187.4 (53.316)**	**<0.001**	100.5 (55.969)	0.077
Phosphate (mmol/l)	9.1 (18.660)	0.627	15.9 (22.591)	0.484
cFGF23 (pg/ml)	**0.14 (0.051)**	**0.007**	**0.21 (0.052)**	**<0.001**
Klotho (pg/ml)	−0.012 (0.015)	0.445	**−0.037 (0.017)**	**0.036**
iPTH (pg/ml)	−0.07 (0.138)	0.614	0.06 (0.162)	0.711
25(OH)D (nmol/l)	0.29 (0.181)	0.111	0.03 (0.186)	0.153
1,25(OH)_2_D (pmol/l)	**−0.34 (0.092)**	**<0.001**	**−0.40 (0.102)**	**<0.001**
CRP (nmol/l)	0.042 (0.057)	0.464	0.064 (0.082)	0.437
TNFa (pg/ml)	0.04 (0.913)	0.965	1.07 (0.723)	0.143
IL6 (pg/ml)	**1.57 (0.721)**	**0.031**	0.73 (0.686)	0.404
IL10 (pg/ml)	−10.24 (8.029)	0.207	−0.34 (2.139)	0.874
Iron (μmol/l)	−0.28 (0.563)	0.619	−1.08 (0.610)	0.080
Hepcidin (ng/ml)	<0.001(0.0002)	0.078	<0.001 (0.0003)	0.258

^a^β-coefficients and associated *P*-values from univariate linear regression analysis; Dependent variables are given in bold in grey rows; independent variable are given in open rows; significant (*P* < 0.05) associations with independent variables are indicated in bold. For abbreviations see Table 1.

**Table 3:** Predictors of c-terminal and intact FGF23 at baseline and 12 months^a^ in multivariate regression models

**A.**	Baseline		12 months	
	β-coefficient (SE)	*P* value	β-coefficient (SE)	*P* value
cFGF23 (RU/ml)				
eGFR (ml/min/1.73 m^2^)	**−1.07 (0.27)**	**<0.001**	**−0.83 (0.26)**	**0.002**
iFGF23 (pg/ml)	–	–	**0.74 (0.22)**	**0.001**
Hepcidin (ng/ml)	–	–	**−0.001 (0.001)**	**0.007**
iFGF23 (pg/ml)				
Calcium_ALB_ (mmol/l)	**157.5 (50.0)**	**0.002**	–	–
cFGF23 (pg/ml)	**0.10 (0.05)**	**0.034**	**0.17 (0.05)**	**0.001**
1,25(OH)_2_D (pmol/l)	**−0.223 (0.091)**	**0.017**	**−0.319 (0.099)**	**0.002**
B.	Baseline	12 months	Baseline	12 months
	β-coefficient (SE)	*P* value	β-coefficient (SE)	*P* value
cFGF23 (RU/ml)				
eGFR (ml/min/1.73 m^2^)	−1.07 (0.27)	<0.001	−1.15 (0.26)	<0.001
Hepcidin (ng/ml)	–	–	−0.001 (0.001)	0.029
iFGF23 (pg/ml)				
Calcium_ALB_ (mmol/l)	152.68 (51.31)	0.004	–	–
1,25(OH)_2_D (pmol/l)	−0.28 (0.09)	0.002	−0.40 (0.10)	<0.001

^a^β-coefficients and associated *P*-values from multivariate regression analysis with (A) iFGF23 or cFGF23 and (B) after removal of iFGF23 or cFGF23 as independent variables. Dependent variables are given in bold in grey rows; independent variables are given in open rows. For abbreviations see Table 1.

Significant predictors of iFGF23 were eGFR, cFGF23, 1,25(OH)_2_D, albumin adjusted calcium and IL6 (all *P* < 0.05) and a tendency for hepcidin (*P* = 0.078). No significant associations were found with other markers of inflammation and iron status (Table 2). In a multivariate model, cFGF23, 1,25(OH)_2_D and adjusted calcium remained significant (total *R*^2^ = 31%). Removal of cFGF23 as an independent variable did not materially change these findings (Table 3).

At 12 months, predictors of cFGF23 were eGFR, iFGF23, PTH, 1,25(OH)_2_D, albumin adjusted calcium and IL6 and there was a tendency of significance for hepcidin (*P* = 0.077) and CRP (*P* = 0.070) (Table 2). In a multivariate model, eGFR, iFGF23 and hepcidin were significant (total *R*^2^ = 37%). Removal of iFGF23 as independent variable did not materially change these findings (Table 3).

Predictors of iFGF23 were eGFR, cFGF23, Klotho and 1,25(OH)_2_D, and a tendency for iron (*P* = 0.08), of which cFGF23 and 1,25(OH)_2_D were significant in the multivariate model (total *R*^2^ = 30%). After removal of cFGF23 as independent variable, 1,25(OH)_2_D remained significant.

## Discussion

In this *post-hoc* analysis of a 12-month double-blind RCT with vitamin D_3_ in older people, we showed that baseline plasma concentration of cFGF23, iFGF23, PTH, SOST and TNFα were higher, while Klotho, 1,25(OH)_2_D and iron were lower in the group with early renal impairment (CKDG3a/b) compared to the group with normal renal function. After supplementation, only cFGF23, 25(OH)D and IL6 differed between groups. The response to supplementation differed by eGFR category; a significant decrease in TNFα and increase in iron was only found in the CKDG3a/b group. In the group with normal renal function, plasma P decreased and cFGF23, iFGF23 and P1NP increased. A significant increase in 25(OH)D and IL10 and a decrease in CTX was found in both groups. In univariate regression analyses both cFGF23 and iFGF23 were predicted by renal function and regulators of calcium and phosphate metabolism. IL6 significantly predicted iFGF23 at baseline and cFGF23 after supplementation, which did not remain significant in multivariate models. Post-supplementation hepcidin, together with eGFR predicted cFGF23 in the multivariate models.

The alterations in regulators of calcium, phosphate and vitamin D metabolism found in the group with early renal impairment (predominantly CKDG3a) at baseline are consistent with changes reported in more advanced stages of CKD [12, 41, 42]. The pro-inflammatory cytokines TNFα and IL6 were significantly or tended to be higher in the group with renal impairment, indicating a state of higher chronic inflammation. Most participants (86%), regardless of their renal function, had CRP values within the reference range (<47.6 nmol/l) [43], indicating absence of acute inflammation. Our findings also suggest that a decline in iron status occurs from early renal impairment, although this was not accompanied by higher hepcidin concentrations. This may potentially only occur when iron falls below the threshold of deficiency (<5.8 μmol/l for the method used), observed in very few study participants. Recent studies suggest that systemic inflammation and iron deficiency upregulate FGF23 transcription [4, 44, 45] and cleavage into C-terminal FGF23, leading to a mild increase in iFGF23 but a significant increase in cFGF23 [46–48]. Also in our study cFGF23 was proportionally higher in the CKDG3a compared to the CKDG1 group (baseline iFGF23 to cFGF23 ratio 0.72 and 0.98, respectively; *P* < 0.01). The altered iFGF23 to cFGF23 ratio together with lower Klotho expression potentially modulates iFGF23 sensitivity [49].

Our data indicate that the effect of vitamin D_3_ supplementation on inflammation and iron status may be influenced by renal function. Supplementation significantly decreased plasma TNFα and increased iron concentrations only in the group with renal impairment, to values comparable to those in the group with normal renal function. IL10 increased in both groups. These results are similar to the reported effects of pro-inflammatory and anti-inflammatory cytokines to vitamin D supplementation and/or VDR activation in *in vitro* models and in human studies mostly including participants with conditions associated with increased inflammation [25–27, 30, 31, 50, 51]. It has been suggested that an increase in iron status after vitamin D supplementation is mediated through a decline in IL6 and TNFα and an increase in IL10 [8, 21, 29, 52, 53]. This possibly explains that an increase in iron status was only seen in the group with renal impairment. Although a reduction in hepcidin may be expected to be observed simultaneously and has been observed after a bolus of vitamin D [5, 52, 54–56], in our study hepcidin did not significantly change.

A significant increase in iFGF23 and cFGF23 after vitamin D_3_ supplementation was only found in the group with normal renal function, but the data distribution in group with renal impairment was wide, limiting the statistical power to detect a change. A corresponding decrease in plasma P was found in the group with normal renal function. It may be speculated this was the result of differences in the before-mentioned iFGF23 sensitivity. The mechanisms of the increase in FGF23 with vitamin D_3_ supplementation remains to be elucidated, but has been reported before by our group and others [14, 33]. The health impact of an increase in FGF23 without CKD, hyperphosphatemia and elevated PTH is unclear.

Vitamin D_3_ supplementation did not substantially change predictors of cFGF23 and iFGF23. Both cFGF23 and iFGF23 were predicted by each other, by renal function and regulators of calcium and phosphate metabolism. Associations with plasma IL6 were significant for cFGF23 (12 month) and iFGF23 (at baseline) and there were tendencies (*P* < 0.1) of significant associations with CRP and markers of iron status. In multivariate models with eGFR, hepcidin significantly predicted cFGF23 at 12 months. These data provide limited evidence that in a small cohort of older people with an eGFR ranging from normal to early renal impairment, inflammatory factors and iron status are determinants of plasma iFGF23 and cFGF23 [7, 8, 46, 47] although these variables explained a minor part of variance.

This study has several limitations. The VDOP study included relatively healthy older adults and excluded those with an eGFR <30 ml/min/1.73 m^2^ and known renal disease at screening. Albuminuria was not considered. The underlying causes of impaired renal function in participants in this study are unknown and may be expected to be heterogenous. As a result, the bone phenotype may also be of a heterogenous nature. We did not measure other markers of iron status or metabolism e.g. ferritin, transferrin or total iron-binding capacity, due to limitations in available sample types. We did not collect data allowing the assessment of dietary iron intake. Therefore, it cannot excluded that group differences in iron status were explained by nutrient intake rather altered iron homeostasis or can we distinguish between absolute and functional low iron supply [1]. Power for statistical analyses was limited due to the small group sizes. The absence of a placebo group [38] (as per guidelines for pharmaceutical trials, a group receiving standard care, i.e. the population RNI was included) did not allow to account for changes unrelated to the intervention (i.e. ageing or secular trends).

## Conclusions

In this community-dwelling cohort of older people considered to be generally healthy, a substantial proportion of participants had undetected early renal impairment. This study showed that early renal impairment (CKDG3a/b) was associated with alterations in PTH, FGF23, vitamin D, inflammation, iron status and SOST, factors know to be associated with negative consequences for bone health and the development of CKD-MBD. After supplementation, few differences between the group with impaired and normal renal function remained. However, our data suggest an altered response in FGF23 and bone metabolism to vitamin D_3_ supplementation with early renal impairment. Predictors of cFGF23 and iFGF23 were eGFR and regulators of calcium and phosphate metabolism. Inflammatory factors and iron status weakly predicted plasma iFGF23 and cFGF23.

This study identified changes in the renal bone-axis occurring before patients are clinically monitored. Diagnosis in the early stages of renal impairment may provide opportunities for the prevention and progression of renal disease and CKD-MBD.

## Supplementary Material

SupplementaryData_AA-23-1371_R2_mod_03_afae096

## Data Availability

Primary data and outcomes were published per EUDRACT protocol.
