# Supplementary material for: Alterations in regulators of the renal-bone axis, inflammation and iron status in older people with early renal impairment and the effect of vitamin D supplementation
Source: Age Ageing. 2024 May 20;53(5):afae096. doi: 10.1093/ageing/afae096 (PMC11106582; doi:10.1093/ageing/afae096)
Supplement: SupplementaryData_AA-23-1371_R2_mod_03_afae096 [file supplementarydata_aa-23-1371_r2_mod_03_afae096.docx]

**Predictors of Mortality Shortly After Entering a Long-Term Care Facility**

**SUPPLEMENTARY DATA**

**Supplementary Table 1.** Individual and facility characteristics by mortality status at 90 days after entry into a long-term care facility (not already included in Table 1).

|  | ***Total N*** |  | ***Alive at 90 days*** | ***Died within 90 days*** |
| --- | --- | --- | --- | --- |
| **Number participants** | **116192** |  | **105282 (90.6)** | **10910 (9.4)** |
| **Age group, years** |  |  |  |  |
| 65-84 | 57128 |  | 52065 (91.1) | 5063 (8.9) |
| 85+ | 59064 |  | 53217 (90.1) | 5847 (9.9) |
| **Has partner** | 43704 |  | 38887 (89.0) | 4817 (11.0) |
| **Delirium** | 6608 |  | 5763 (87.2) | 845 (12.8) |
| **Depression** | 60667 |  | 54856 (90.4) | 5811 (9.6) |
| **History of skin disease** | 11435 |  | 10060 (88.0) | 1375 (12.0) |
| **ROSA Frailty Index Score^1^** |  |  |  |  |
| 0 to <0.1 | 450 |  | 432 (96.0) | 18 (4.0) |
| ≥0.1 to <0.2 | 11315 |  | 10651 (94.1) | 664 (5.9) |
| ≥0.2 to <0.3 | 60238 |  | 55054 (91.4) | 5184 (8.6) |
| ≥0.3 | 31841 |  | 28078 (88.2) | 3763 (11.8) |
| **Number of RxRisk-V Co-morbidities** |  |  |  |  |
| 0-1 | 9069 |  | 8462 (93.3) | 607 (6.7) |
| 2-3 | 21024 |  | 19493 (92.7) | 1531 (7.3) |
| 4-5 | 32190 |  | 29465 (91.5) | 2725 (8.5) |
| 6-7 | 29300 |  | 26407 (90.1) | 2893 (9.9) |
| 8+ | 24609 |  | 21455 (87.2) | 3154 (12.8) |
| **Any respite care prior to permanent long term care facility entry** | 64832 |  | 59632 (92.0) | 5200 (8.0) |
| **In respite care, year before permanent long term care facility entry** | 62106 |  | 57159 (92.0) | 4947 (8.0) |

ROSA=Registry of Senior Australians.

1. Missing data: ROSA Frailty Index Score n=12348 (10.6%).

**Supplementary Table 2.** *M*edication factors within 90 days prior to permanent long-term facility entry by mortality status at 90 days (not already included in Table 1).

|  | ***Total N*** |  | ***Alive at 90 days*** | ***Died within 90 days*** |
| --- | --- | --- | --- | --- |
| **Number participants** | **116192** |  | **105282 (90.6)** | **10910 (9.4)** |
| **Sedative load score rating** |  |  |  |  |
| 0-1 | 61768 |  | 56688 (91.8) | 5080 (8.2) |
| 2-3 | 33620 |  | 30312 (90.2) | 3308 (9.8) |
| 4-5 | 14265 |  | 12614 (88.4) | 1651 (11.6) |
| 6-7 | 4835 |  | 4218 (87.2) | 617 (12.8) |
| 8+ | 1704 |  | 1450 (85.1) | 254 (14.9) |
| **Specific medications (ATC codes)^1^** |  |  |  |  |
| Proton pump inhibitors (A02BC) | 52365 |  | 46625 (89.0) | 5740 (11.0) |
| Propulsives (A03FA) | 9729 |  | 8104 (83.3) | 1625 (16.7) |
| Osmotically acting laxatives (A06AD) | 25886 |  | 22930 (88.6) | 2956 (11.4) |
| Biguanides (A10BA) | 11508 |  | 10508 (91.3) | 1000 (8.7) |
| Sulfonylureas (A10BB) | 7509 |  | 6715 (89.4) | 794 (10.6) |
| Potassium salts (A12BA) | 6923 |  | 5930 (85.7) | 993 (14.3) |
| Vitamin K antagonists (B01AA) | 11989 |  | 10528 (87.8) | 1461 (12.2) |
| Heparin group (B01AB) | 7265 |  | 6322 (87.0) | 943 (13.0) |
| Platelet aggregation (B01AC) inhibitors excl. heparin | 34439 |  | 31049 (90.2) | 3390 (9.8) |
| Digitalis glycosides (C01AA) | 8830 |  | 7594 (86.0) | 1236 (14.0) |
| Organic nitrates (C01DA) | 10254 |  | 8943 (87.2) | 1311 (12.8) |
| Sulfonamides, plain (C03CA) | 28957 |  | 24839 (85.8) | 4118 (14.2) |
| Aldosterone antagonists (C03DA) | 6729 |  | 5558 (82.6) | 1171 (17.4) |
| Beta blocking agents, selective (C07AB) | 28944 |  | 25689 (88.8) | 3255 (11.2) |
| Dihydropyridine derivatives (C08CA) | 18261 |  | 16760 (91.8) | 1501 (8.2) |
| ACE inhibitors, plain (C09AA) | 25271 |  | 22917 (90.7) | 2354 (9.3) |
| Angiotensin II receptor blockers (ARBs), plain (C09CA) | 20707 |  | 19056 (92.0) | 1651 (8.0) |
| Angiotensin II receptor blockers (ARBs) and diuretics (C09DA) | 6219 |  | 5792 (93.1) | 427 (6.9) |
| HMG CoA reductase inhibitors (C10AA) | 46690 |  | 42625 (91.3) | 4065 (8.7) |
| Glucocorticoids (H02AB) | 11809 |  | 9751 (82.6) | 2058 (17.4) |
| Thyroid hormones (H03AA) | 9321 |  | 8408 (90.2) | 913 (9.8) |
| Penicillins with extended spectrum (J01CA) | 6493 |  | 5743 (88.4) | 750 (11.6) |
| Combinations of penicillins, incl. beta-lactamase inhibitors (J01CR) | 9927 |  | 8504 (85.7) | 1423 (14.3) |
| First-generation cephalosporins (J01DB) | 20237 |  | 17878 (88.3) | 2359 (11.7) |
| Trimethoprim and derivatives (J01EA) | 8400 |  | 7585 (90.3) | 815 (9.7) |
| Preparations inhibiting uric acid production (M04AA) | 6093 |  | 5255 (86.2) | 838 (13.8) |
| Bisphosphonates (M05BA) | 6531 |  | 5992 (91.7) | 539 (8.3) |
| Bisphosphonates, combinations (M05BB) | 5871 |  | 5390 (91.8) | 481 (8.2) |
| Natural opium alkaloids (N02AA) | 22935 |  | 19735 (86.0) | 3200 (14.0) |
| Oripavine derivatives (N02AE) | 8119 |  | 7204 (88.7) | 915 (11.3) |
| Anilides (N02BE) | 55182 |  | 49853 (90.3) | 5329 (9.7) |
| Other analgesics and antipyretics (N02BG) | 7031 |  | 6273 (89.2) | 758 (10.8) |
| Dopa and dopa derivatives (N04BA) | 6971 |  | 6424 (92.2) | 547 (7.8) |
| Diazepine, oxazepine, thiazepine and oxepine anti-psychotics (N05AH) | 6741 |  | 6218 (92.2) | 523 (7.8) |
| Other antipsychotics (N05AX) | 10751 |  | 9795 (91.1) | 956 (8.9) |
| Benzodiazepine derivatives (N05BA) | 11879 |  | 10708 (90.1) | 1171 (9.9) |
| Benzodiazepine derivatives (N05CD) | 14948 |  | 13245 (88.6) | 1703 (11.4) |
| Tricyclic antidepressants (N06AA) | 6303 |  | 5634 (89.4) | 669 (10.6) |
| Selective serotonin reuptake inhibitors (N06AB) | 20109 |  | 18481 (91.9) | 1628 (8.1) |
| Other antidepressants (N06AX) | 16423 |  | 14964 (91.1) | 1459 (8.9) |
| Anticholinesterases (N06DA) | 12036 |  | 11342 (94.2) | 694 (5.8) |
| Selective beta-2-adrenoreceptor agonists (R03AC) | 12744 |  | 10864 (85.2) | 1880 (14.8) |
| Adrenergic in combination with corticosteroids or other drugs, excl. anticholinergics (R03AK) | 12623 |  | 10943 (86.7) | 1680 (13.3) |
| Inhaled anticholinergics (R03BB) | 11133 |  | 9412 (84.5) | 1721 (15.5) |
| Prostaglandin analogues (S01EE) | 10512 |  | 9554 (90.9) | 958 (9.1) |
| Other ophthalmologicals (S01XA) | 12142 |  | 11054 (91.0) | 1088 (9.0) |

ATC= Anatomical, Therapeutic and Chemical classification codes.

**Supplementary Table 3.** *Hospital healthcare* factors one year prior^1^ to permanent long-term care facility entry by mortality status at 90 days (not already included in Table 2).

|  | ***Total N*** |  | ***Alive at 90 days*** | ***Died within 90 days*** |
| --- | --- | --- | --- | --- |
| **Number participants** | **116192** |  | **105282 (90.6)** | **10910 (9.4)** |
| **No. unplanned hospitalisations** |  |  |  |  |
| 0 | 36053 |  | 34097 (94.6) | 1956 (5.4) |
| 1 | 41383 |  | 37585 (90.8) | 3798 (9.2) |
| 2-4 | 34321 |  | 29962 (87.3) | 4359 (12.7) |
| 5+ | 4435 |  | 3638 (82.0) | 797 (18.0) |
| **LOS unplanned hospitalisations, days** |  |  |  |  |
| 0 | 36080 |  | 34122 (94.6) | 1958 (5.4) |
| 1-30 | 43042 |  | 38742 (90.0) | 4300 (10.0) |
| 31-90 | 30051 |  | 26238 (87.3) | 3813 (12.7) |
| 91+ | 7019 |  | 6180 (88.0) | 839 (12.0) |
| **No. potentially preventable hospitalisations** |  |  |  |  |
| 0 | 96063 |  | 88014 (91.6) | 8049 (8.4) |
| 1 | 14691 |  | 12828 (87.3) | 1863 (12.7) |
| 2-4 | 4969 |  | 4073 (82.0) | 896 (18.0) |
| 5+ | 469 |  | 367 (78.3) | 102 (21.7) |
| **LOS potentially preventable hospitalisations, days** |  |  |  |  |
| 0 | 96069 |  | 88020 (91.6) | 8049 (8.4) |
| 1-30 | 15283 |  | 13214 (86.5) | 2069 (13.5) |
| 31-90 | 4252 |  | 3553 (83.6) | 699 (16.4) |
| 91+ | 588 |  | 495 (84.2) | 93 (15.8) |
| **No. ED presentations** |  |  |  |  |
| 0 | 32296 |  | 30410 (94.2) | 1886 (5.8) |
| 1 | 34475 |  | 31501 (91.4) | 2974 (8.6) |
| 2-4 | 40278 |  | 35656 (88.5) | 4622 (11.5) |
| 5+ | 9143 |  | 7715 (84.4) | 1428 (15.6) |
| **Unplanned hospitalisations (30 days prior)** | 42400 |  | 36475 (86.0) | 5925 (14.0) |
| **Potentially preventable hospitalisations (30 days prior)** | 6797 |  | 5645 (83.1) | 1152 (16.9) |
| **ED presentation (30 days prior)** | 23473 |  | 20033 (85.3) | 3440 (14.7) |

LOS=Length of Stay. ED=Emergency Department. No=Number.

1. All variables are ascertained using the one-year period before entry into permanent long term care unless otherwise specified (i.e. 30 days).

**Supplementary Table 4.** *Medicare Benefits Schedule-subsidised services healthcare factors* one year prior to permanent long-term care facility entry by mortality status 90 days (not already included in Table 2).

| ***Service (MBS item group)^1^*** | ***Total N*** |  | ***Alive at 90 days*** | ***Died within 90 days*** |
| --- | --- | --- | --- | --- |
| **Number participants** | **116192** |  | **105282 (90.6)** | **10910 (9.4)** |
| **GP management plans etc (MBS group A15) 1 or more** |  |  |  |  |
| 0 | 52033 |  | 47321 (90.9) | 4712 (9.1) |
| 1+ | 64159 |  | 57961 (90.3) | 6198 (9.7) |
| **Optometrical services (A10)** |  |  |  |  |
| 0 | 76292 |  | 68827 (90.2) | 7465 (9.8) |
| 1 | 28856 |  | 26297 (91.1) | 2559 (8.9) |
| 2-4 | 10518 |  | 9681 (92.0) | 837 (8.0) |
| 5+ | 526 |  | 477 (90.7) | 49 (9.3) |
| **GP after-hours attendance (A22)** |  |  |  |  |
| 0 | 77219 |  | 70013 (90.7) | 7206 (9.3) |
| 1 | 20459 |  | 18622 (91.0) | 1837 (9.0) |
| 2-4 | 13562 |  | 12207 (90.0) | 1355 (10.0) |
| 5+ | 4952 |  | 4440 (89.7) | 512 (10.3) |
| **GP health assessments (A14)** |  |  |  |  |
| 0 | 79249 |  | 71621 (90.4) | 7628 (9.6) |
| 1 | 34185 |  | 31145 (91.1) | 3040 (8.9) |
| 2 | 2667 |  | 2432 (91.2) | 235 (8.8) |
| 3+ | 91 |  | 84 (92.3) | 7 (7.7) |
| **Urgent attendance after hours (A11)** |  |  |  |  |
| 0 | 88322 |  | 80376 (91.0) | 7946 (9.0) |
| 1 | 17676 |  | 15906 (90.0) | 1770 (10.0) |
| 2-4 | 8868 |  | 7838 (88.4) | 1030 (11.6) |
| 5+ | 1326 |  | 1162 (87.6) | 164 (12.4) |
| **Geriatric medicine (A28)** |  |  |  |  |
| 0 | 106153 |  | 95971 (90.4) | 10182 (9.6) |
| 1 | 6610 |  | 6129 (92.7) | 481 (7.3) |
| 2-4 | 3374 |  | 3135 (92.9) | 239 (7.1) |
| 5+ | 55 |  | 47 (85.5) | 8 (14.5) |
| **Other non-referred attendances to which no other item applies (A02)** |  |  |  |  |
| 0 | 106182 |  | 96329 (90.7) | 9853 (9.3) |
| 1 | 5329 |  | 4778 (89.7) | 551 (10.3) |
| 2-4 | 2869 |  | 2539 (88.5) | 330 (11.5) |
| 5+ | 1812 |  | 1636 (90.3) | 176 (9.7) |
| **Medication management review (A17)** |  |  |  |  |
| 0 | 109449 |  | 99117 (90.6) | 10332 (9.4) |
| 1 | 6640 |  | 6071 (91.4) | 569 (8.6) |
| 2-4 | 103 |  | 94 (91.3) | 9 (8.7) |

MBS=Medicare Benefits Schedule. GP= General Practitioners.

1. Only showing services with >=5% prevalence.

**Supplementary Table 5.** Main causes of mortality in the cohort within 90 days of entry into a permanent long-term care in a facility.

| **ICD-10-AM Group: Descriptions** | **N** | **%** |
| --- | --- | --- |
| Deaths, total^1^ | 10787 | 100 |
| I00-I99: Diseases of the circulatory system | 3689 | 34.2 |
| I21.9: Acute myocardial infarction, unspecified | 656 | 17.8 |
| I25.9: Chronic ischaemic heart disease, unspecified | 642 | 17.4 |
| I64: Stroke, not specified as haemorrhage or infarction | 504 | 13.7 |
| Other | 1887 | 51.2 |
| C00-D48: Neoplasms | 2294 | 21.3 |
| C34.9: Malignant neoplasm of bronchus and lung, unspecified | 375 | 17.1 |
| C61: Malignant neoplasm of prostate | 293 | 13.3 |
| C50.9: Malignant neoplasm of breast, unspecified | 139 | 6.3 |
| Other | 1388 | 63.2 |
| J00-J99: Diseases of the respiratory system | 1257 | 11.7 |
| J44.9: Chronic obstructive pulmonary disease, unspecified | 339 | 27.0 |
| J44.0: Chronic obstructive pulmonary disease with acute lower  respiratory infection (excl influenza) | 185 | 14.7 |
| J18.9: Pneumonia, unspecified | 179 | 14.2 |
| Other | 554 | 44.1 |
| F00-F99: Mental and behavioural disorders | 806 | 7.5 |
| F03: Unspecified dementia | 636 | 78.9 |
| F01.9: Vascular dementia, unspecified | 126 | 15.6 |
| F05.9: Delirium, unspecified | 13 | 1.6 |
| Other | 31 | 3.8 |
| G00-G99: Diseases of the nervous system | 687 | 6.4 |
| E00-E99: Endocrine, nutritional and metabolic diseases | 576 | 5.3 |
| K00-K99: Diseases of the digestive system | 340 | 3.2 |
| V00-Y99: External causes of morbidity and mortality | 332 | 3.1 |
| N00-N99: Diseases of the genitourinary system | 296 | 2.7 |
| A00-B99: Certain infectious and parasitic diseases | 236 | 2.2 |
| Other | 274 | 2.5 |
|  |  |  |

ICD-10-AM = International Statistical Classification of Diseases and Related Health Problems, Tenth Revision, Australian Modification.

1. For 123 individuals, no cause of death is specified.

**Supplementary Table 6.** Mortality prediction model coefficients and out of sample validation Harrell C index and 95% confidence intervals.^1^

| **Factors*** | Final Model^2^ | Alternative Model^3^ | Base Model^4^ |
| --- | --- | --- | --- |
| Baseline 90 day survival, S_0_(90 day) | 0.985231 | 0.983181 | 0.923198 |
| **Individual factors** |  |  |  |
| Men vs women | 0.462019 | 0.460944 | 0.520420 |
| Age in decades (centered at 85 years) | 0.295088 | 0.292345 | 0.180061 |
| Dementia | -0.133493 | -0.112860 |  |
| Incontinence | -0.189168 | -0.186680 |  |
| History of falls | -0.204935 | -0.205932 |  |
| History of malnutrition | 0.155301 | 0.145558 |  |
| ADL none vs. ADL low | -1.305501 | -1.275092 |  |
| ADL medium vs. ADL low | 0.640110 | 0.639652 |  |
| ADL high vs. ADL low | 1.689141 | 1.696009 |  |
| BEH medium vs. BEH low | -0.108759 | -0.149405 |  |
| BEH high vs. BEH low | 0.035047 | -0.053922 |  |
| CHC High vs. CHC Low | 0.514229 | 0.401877 |  |
| Respite care (1 year prior) | -0.132691 | -0.110371 |  |
| State: Victoria vs New South Wales | -0.107122 |  |  |
| Major city vs inner regional areas | -0.137833 | -0.128957 |  |
| For-profit vs non-profit facility | 0.119431 | 0.085944 |  |
| Government funded vs non-profit facility | 0.203759 | 0.107348 |  |
| Year: 2013 vs 2015 | 0.303781 |  |  |
| Year: 2014 vs 2015 | 0.180591 |  |  |
| Number of medications, truncated to 13 | 0.044465 | 0.042442 |  |
| Propulsives (for functional gastrointestinal disorders) (A03FA) | 0.381163 | 0.354382 |  |
| Biguanide blood glucose lowering drugs (e.g. metformin) (A10BA) | -0.194873 | -0.197636 |  |
| Potassium supplement (A12BA) | 0.115715 | 0.097603 |  |
| Organic nitrate (cardiac) vasodilators (C01DA) | 0.108173 | 0.118529 |  |
| Sulfonamides, plain (C03CA) | 0.218494 | 0.205770 |  |
| Aldosterone antagonist diuretics (C03DA) | 0.214512 | 0.217720 |  |
| Dihydropyridine calcium channel blockers (C08CA) | -0.113111 | -0.117881 |  |
| ACE inhibitors, plain (C09AA) | -0.134908 | -0.132006 |  |
| Angiotensin II receptor blockers (ARBs), plain (C09CA) | -0.177588 | -0.173844 |  |
| HMG CoA reductase inhibitors (statins) (C10AA) | -0.172687 | -0.168835 |  |
| Glucocorticoids - corticosteroids for systemic use (H02AB) | 0.337637 | 0.334644 |  |
| Bisphosphonates (M05BA) | -0.214768 | -0.195252 |  |
| Natural opium alkaloids (N02AA) | 0.117098 | 0.095373 |  |
| Anilide analgesics (N02BE) | -0.182539 | -0.157748 |  |
| Other analgesics and antipyretics (N02BG) | -0.201300 | -0.252054 |  |
| Dopa and dopa derivative anti-Parkinson drugs (N04BA) | -0.451951 | -0.461270 |  |
| Selective serotonin reuptake inhibitor antidepressants (N06AB) | -0.239743 | -0.243407 |  |
| Other antidepressants (N06AX) | -0.162550 | -0.179086 |  |
| Anticholinesterase anti-dementia drugs (N06DA) | -0.140765 | -0.158503 |  |
| Anticholinergics - inhaled drugs for obstructive airway diseases (R03BB) | 0.147896 | 0.146317 |  |
| Other ophthalmologicals (S01XA) | -0.197646 | -0.195218 |  |
| No. unplanned hospitalisations, truncated to 6 | 0.043136 | 0.050089 |  |
| Unplanned hospitalisations (30 days prior) | 0.238304 | 0.267181 |  |
| Unplanned ED presentation (30 days prior) | 0.166033 | 0.178372 |  |
| Number of GP attendances, truncated to 15 | 0.010943 | 0.011325 |  |
| Three or more services for patients in LTCFs | -0.101290 | -0.103646 |  |
| Complex health conditions high vs. low × Victoria | -0.220879 |  |  |
| Propulsives × age in decades | -0.176922 | -0.175033 |  |
| HMG CoA reductase inhibitors × age in decades | 0.138240 | 0.135931 |  |
| Natural opium alkaloids × age in decades | -0.122549 | -0.126493 |  |
| Number of GP Attendances, truncated to 15 (A01) × age in decades | -0.012030 | -0.013189 |  |
| Harrel C-Index | 0.773 | 0.773 | 0.585 |
| 95% Confidence Interval | 0.765-0.781 | 0.765-0.781 | 0.574-0.596 |

1. The predicted 90 day survival probability can be generated with model coefficients using the formula: Pr(90 day mortality) = 1 - [S_0_(90 day) ]^exp(^**^β^**^.^**^x^**^)^, where **β** is the vector of parameter values shown in the table and **x** is the vector of corresponding covariate values for an individual’s characteristics. Some of the covariate values require transformation, as specified in the first column of this table.
2. Final presented model.
3. Model includes all variables in final presented model except Australian specific variables (state and year).
4. Model includes age and sex only.

**Supplementary Table 7.** Filled-in TRIPOD checklist for Prediction Model Development.

| **Section/Topic** | **Item** | **Checklist Item** | **Page** |
| --- | --- | --- | --- |
| **Title and abstract** | | | |
| Title | 1 | Identify the study as developing and/or validating a multivariable prediction model, the target population, and the outcome to be predicted. | Title |
| Abstract | 2 | Provide a summary of objectives, study design, setting, participants, sample size, predictors, outcome, statistical analysis, results, and conclusions. | Abstract |
| **Introduction** | | | |
| Background and objectives | 3a | Explain the medical context (including whether diagnostic or prognostic) and rationale for developing or validating the multivariable prediction model, including references to existing models. | Introduction para 1-3 |
|  | 3b | Specify the objectives, including whether the study describes the development or validation of the model or both. | Introduction para 4 |
| **Methods** | | | |
| Source of data | 4a | Describe the study design or source of data (e.g., randomized trial, cohort, or registry data), separately for the development and validation data sets, if applicable. | Methods para 1 |
|  | 4b | Specify the key study dates, including start of accrual; end of accrual; and, if applicable, end of follow-up. | Methods para 2-3 |
| Participants | 5a | Specify key elements of the study setting (e.g., primary care, secondary care, general population) including number and location of centres. | Methods para 2 |
|  | 5b | Describe eligibility criteria for participants. | Methods para 2 |
|  | 5c | Give details of treatments received, if relevant. | N/A |
| Outcome | 6a | Clearly define the outcome that is predicted by the prediction model, including how and when assessed. | Methods para 3 |
|  | 6b | Report any actions to blind assessment of the outcome to be predicted. | N/A |
| Predictors | 7a | Clearly define all predictors used in developing or validating the multivariable prediction model, including how and when they were measured. | Methods para 4-8 |
|  | 7b | Report any actions to blind assessment of predictors for the outcome and other predictors. | N/A; uses admin data |
| Sample size | 8 | Explain how the study size was arrived at. | Methods para 2, footnote |
| Missing data | 9 | Describe how missing data were handled (e.g., complete-case analysis, single imputation, multiple imputation) with details of any imputation method. | Methods para 9 |
| Statistical analysis methods | 10a | Describe how predictors were handled in the analyses. | Methods para 9 |
|  | 10b | Specify type of model, all model-building procedures (including any predictor selection), and method for internal validation. | Methods para 9 |
|  | 10d | Specify all measures used to assess model performance and, if relevant, to compare multiple models. | Methods para 9 |
| Risk groups | 11 | Provide details on how risk groups were created, if done. | N/A |
| **Results** | | | |
| Participants | 13a | Describe the flow of participants through the study, including the number of participants with and without the outcome and, if applicable, a summary of the follow-up time. A diagram may be helpful. | Tables 1,2; footnote |
|  | 13b | Describe the characteristics of the participants (basic demographics, clinical features, available predictors), including the number of participants with missing data for predictors and outcome. | Tables 1,2 |
| Model development | 14a | Specify the number of participants and outcome events in each analysis. | Tables 1,2 |
|  | 14b | If done, report the unadjusted association between each candidate predictor and outcome. | Tables 1,2 |
| Model specification | 15a | Present the full prediction model to allow predictions for individuals (i.e., all regression coefficients, and model intercept or baseline survival at a given time point). | Table 3, Suppl. Table 6 |
|  | 15b | Explain how to the use the prediction model. | Suppl. Table 6 footnote |
| Model performance | 16 | Report performance measures (with CIs) for the prediction model. | Results para 3, Fig 1 caption |
| **Discussion** | | | |
| Limitations | 18 | Discuss any limitations of the study (such as nonrepresentative sample, few events per predictor, missing data). | Discussion para 4 |
| Interpretation | 19b | Give an overall interpretation of the results, considering objectives, limitations, and results from similar studies, and other relevant evidence. | Discussion para 2-4 |
| Implications | 20 | Discuss the potential clinical use of the model and implications for future research. | Discussion para 1; Conclusions |
| **Other information** | | | |
| Supplementary information | 21 | Provide information about the availability of supplementary resources, such as study protocol, Web calculator, and data sets. | Suppl. Table 6 footnote |
| Funding | 22 | Give the source of funding and the role of the funders for the present study. | Declaration of Sources of Funding |

1. Initial selection of individuals consists of new LTCF entrants for years 2013-2016, in Australian states of New South Wales, Victoria and South Australia: N = 149058. After retaining only those individuals: aged 65 years at LTCF entry, not Indigenous, not possessing a Department of Veterans Card, had first PBS concession prior to LTCF entry and had their recorded death date (if present) after their recorded LTCF entry date; N = 122802. After further restricting to those individuals not coded for palliative care in their Aged Care Financial Instrument data, N = 116192, which is the final (training) cohort size.

| **A** | 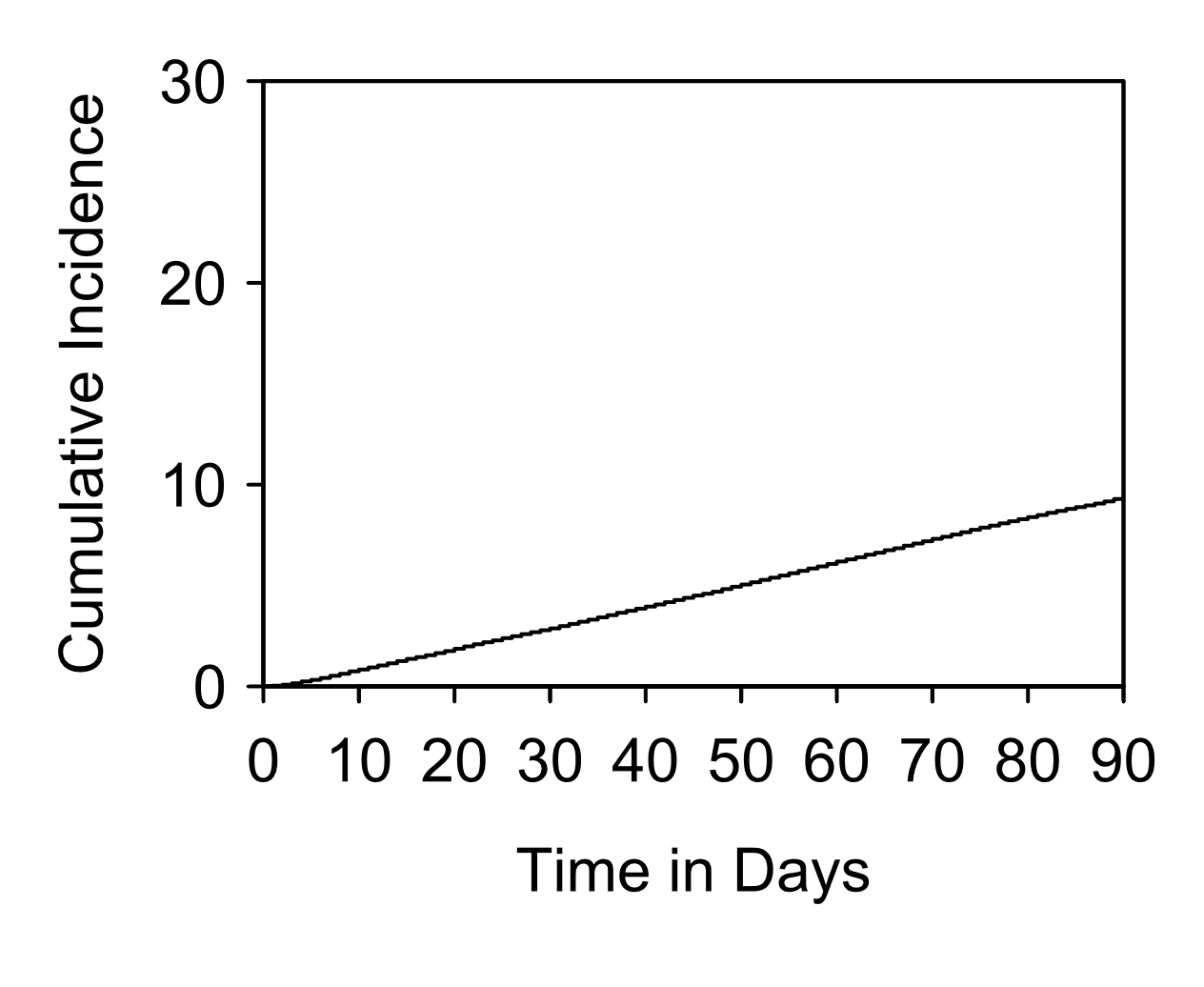 | **B** | 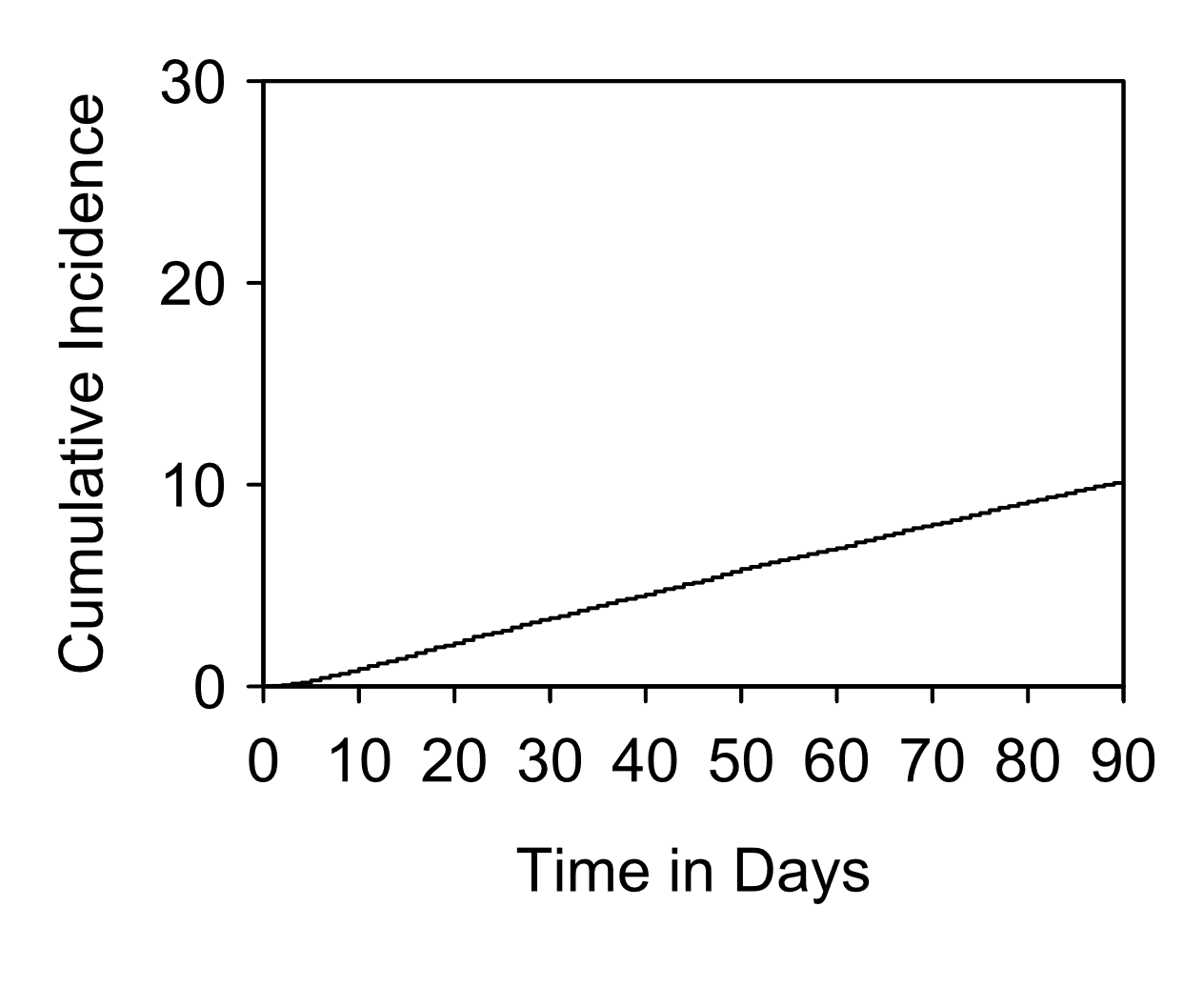 |
| --- | --- | --- | --- |

**Supplementary Figure 1**. Cumulative mortality of cohort to 90 days for (A) main analysis/training cohort and (B) validation set cohort.


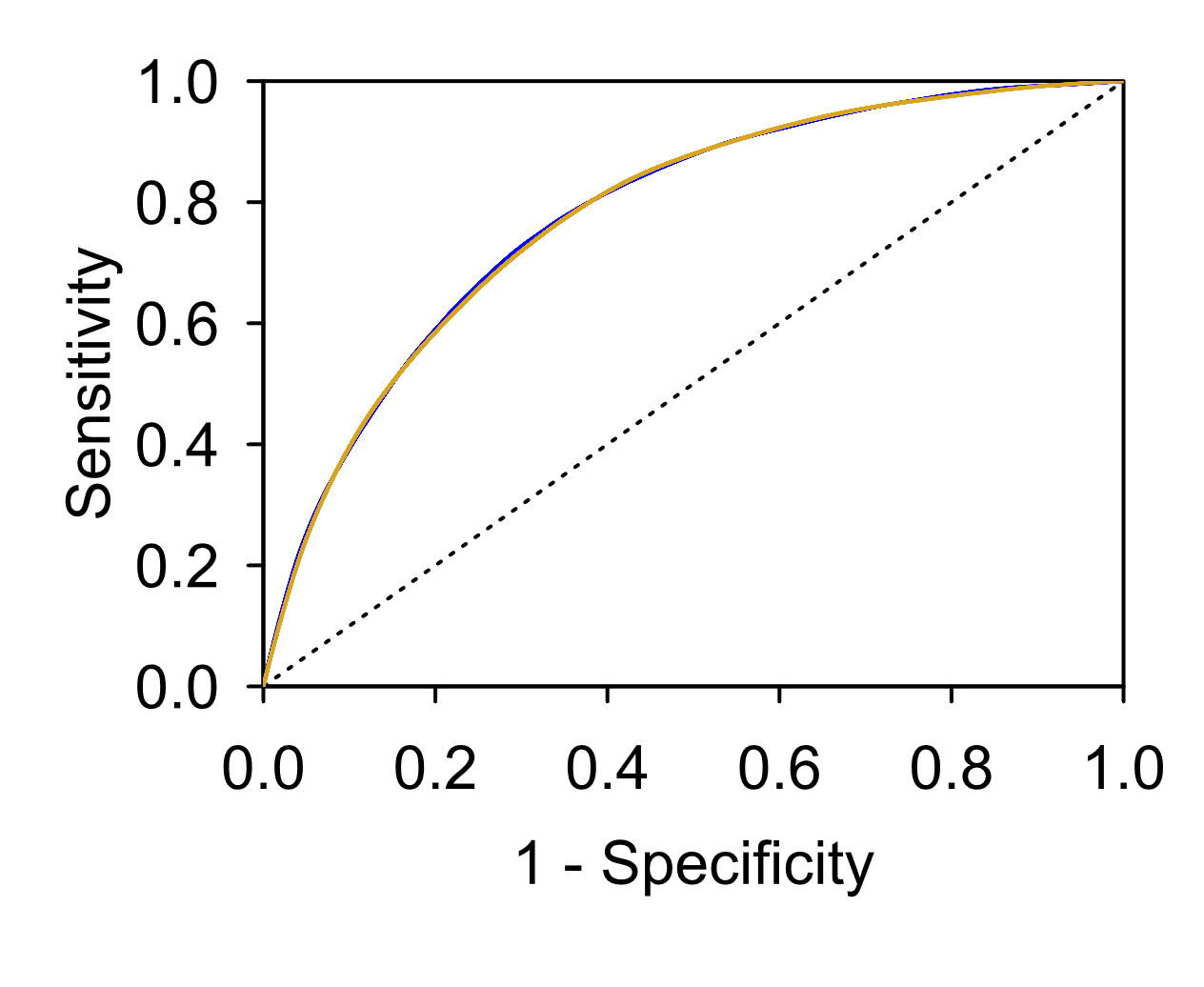


**Supplementary Figure 2**. Receiver Operating Characteristic curves mortality at 90 days after entry into a long term care facility from in-sample 10-fold cross-validation (gold) and from out-of-sample validation (blue).
